# Older Women’s Decision-Making After Receiving Information from a Screening Provider About Breast Cancer Screening Cessation

**DOI:** 10.1007/s11606-025-09931-7

**Published:** 2025-11-18

**Authors:** Jenna Smith, Tara Haynes, Mara A. Schonberg, Nehmat Houssami, Wendy Vincent, Kirsten McCaffery

**Affiliations:** 1 Sydney Health Literacy Lab, Sydney School of Public Health, Faculty of Medicine and Health, The University of Sydney, Sydney, NSW Australia; 2 Department of Medicine, Beth Israel Deaconess Medical Center, Harvard Medical School, Boston, MA USA; 3 The Daffodil Centre, The University of Sydney, a joint venture with the Cancer Council NSW, Sydney, NSW Australia; 4 BreastScreen NSW, Sydney Local Health District, Sydney, NSW Australia

## INTRODUCTION

Guidelines regarding cancer screening cessation vary internationally, and a key priority of screening programs is to ensure potential benefits outweigh harms. In Australia, invitations to screening through the national BreastScreen program cease after age 74, but women can continue to access free screening if desired.^1^ In an online randomized controlled trial of women aged 70–74, information accompanying BreastScreen’s final letter explaining the recommendations increased informed breast screening decision-making, including increased knowledge that the potential harms outweigh benefits for women ≥ 75 years (control: 24%, intervention: 64%).^2,3^ However, there is limited understanding of how women respond to receiving such information. We qualitatively examined older women’s free-text reasons for their breast screening decisions after receiving evidence-based information.

## METHODS

Women aged 70–74 years were recruited online through Qualtrics, a social research company partnering with multiple survey panels to recruit individuals who have consented to receive surveys. Participants read a hypothetical scenario where they received a final BreastScreen letter reporting no abnormalities on their mammogram.^2^ They were randomized to receive: 1) no rationale for cessation (control); 2) printed-text rationale (e.g., downsides of screening may outweigh benefits after age 74); or 3) animation video rationale (see full methods^2^). Ethical approval was obtained from The University of Sydney’s Human Research Ethics Committee (2022/809).

Participants rated their screening intentions beyond 74 years from 1 (definitely will) to 5 (definitely will not), and were asked “Why do you say that?”. Here, we analyzed responses from intervention groups only (groups 2 and 3) to understand how the information provided impacted women’s decision-making.

We used qualitative content analysis in two phases. In Phase 1, one investigator (JS) developed codes from 30% of responses, which another investigator (TH) independently coded (κ = 0.864). Subsequently, the final coding framework was established through discussion and clinical/content expertise, then TH coded all responses and synthesized themes in discussion with a third author (KM). Synthesizing women’s reasons revealed distinct decision-maker types, similar to older adults’ deprescribing decision-making.^4^ In Phase 2, JS and TH independently applied defined typologies to the data, with almost perfect agreement (κ = 0.866). Discrepancies were resolved through consensus.

## RESULTS

Of 254 intervention arm participants, 224 answered the open-ended question. The mean age was 71.9 years; 50.8% reported an education level of high school or less, and 87.8% reported having been screened within the past 2 years.

**Figure 1:**
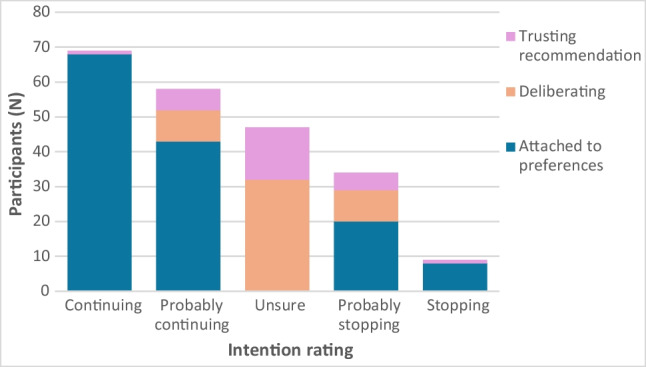
Distribution of intention ratings for each typology.

We observed three dominant typologies (*n* = 217, Table 1) including women who 1) were *attached to prior preferences* (*n* = 139; 64%), driven by screening attitudes or perceived risk regardless of information presented, mostly intending to continue screening; 2)* deliberated* (*n* = 50; 23%), citing intervention content or perceived benefits/risks; or 3)* trusted doctor or BreastScreen recommendations* (*n* = 28; 13%) and were less active decision-makers. The typologies were not mutually exclusive, with some overlapping trends. Figure 1 highlights the distribution of intention ratings within each typology.

**Table 1:** Typologies of Older Women’s Decision-Making

Reasoning	Explanation	Example quote
1. Attached to prior preferences (*N* = 139)		
Strong positive attitudes to screening	Belief in screening, age is irrelevant, preference to continue	“I believe that regular screening is important for all women, regardless of age, and that women of all ages should take advantage of the benefits of early detection.”
Negative attitudes to screening	Do not feel it is necessary, bad previous experiences	“Not necessary for me”
Higher perceived risk of breast cancer	Often due to family history perceive self at higher risk	“My Aunt (mothers sister) was diagnosed with breast cancer after 74 & had the breast removed. So I will probably continue for a couple of more years.”
Lower perceived risk of breast cancer	Lower individual perceived risk often due to no family history or no prior issues	“Never had any issues so will take my chances”
2. Deliberating or considering information (*N* = 50)		
Perceived benefit from continuing	Prefer to continue due to good health, or still prefer to find a slow growing breast cancer	“I may change my mind but at this stage if I have no other health issues I think it would be a sensible thing to do.”
Limited perceived benefit from continuing	Prefer to stop due to slow growing nature of breast cancer, less chance of developing fast growing cancer, having other health problems	“Due to the fact that other health problems arise and the fact that cancers are much slower in advanced age.”
Explicit reference to intervention information	Contemplating content from the intervention	“After watching this video I’m not as sure as I used to be. Before watching I would have certainly gone.”
Further information/advice desired	Explicitly expressing desire for more detailed information	“Would like to read more about it”
3. Trusting doctor or BreastScreen recommendation (*N* = 28)		
No longer recommended	Will stop unless told otherwise, no longer being invited means no need to continue	“As recommendations for screening are not necessary over the age of 74”
Defers to doctor	Unsure and wants to seek a doctor’s recommendation	“I will consult my GP to find out if it is recommended.”
Taking a diagnostic/symptomatic approach	Will let go of screening; monitor for symptoms. Will go to doctor if any particular concerns	“I will probably just see how I go and consult with my doctor if I notice any changes”

This table reports the content analysis of responses from participants in the intervention groups only (printed-text format of information, *N* = 132; animation video format of information, *N* = 122; total *N* = 254)

Responses from participants allocated to the control group (*N* = 122; no information) were excluded

Of the 254 participants who received an intervention, 30 participants reported no reason for their intention rating, so were excluded from the content analysis. *Characteristics of respondents and non-respondents did not differ*

*N* = 7 did not fit typologies

## DISCUSSION

We found three typologies of older women responding to information about breast screening cessation, which can inform multi-level efforts needed to support older women’s decision-making.^5^

Although women attached to prior preferences may not noticeably incorporate new information in their decisions, there is value in providing the option of shared decision-making. In particular, many older women hold beliefs regarding the rationale for recommendations (e.g., perceived ageism, government cost-saving)^6^ and messaging about stopping breast screening needs to happen over time from multiple sources.^3^ If women then desire it, more detailed, accessible shared decision-making resources can supplement this messaging (e.g., patient decision aids). Likewise, tools can support clinicians to provide individualized support so women can have greater trust and understanding of the recommendation and the evidence that informed it.

Limitations of this study include social desirability bias and the hypothetical scenario used. We also assessed intentions about screening beyond 74 years in women aged 70–74 years; their intentions could change after age 74. Finally, our sample may not be representative of women of this age in Australia who would typically attend the nationally funded breast screening services (e.g., underrepresentation of culturally and linguistically diverse women). Future research will refine and test the interventions in a clinical setting with more diverse samples.

Screening services and trusted cancer organizations should deliver brief messaging to explain their recommendations, in addition to increasing the accessibility of shared decision-making resources for women and clinicians.

## Data Availability

Data are available upon reasonable request. De-identified data will be made available upon request to anyone wishing to access it who provides a methodologically sound proposal to the principal investigator. The contact detail of the principal investigator is jenna.smith@sydney.edu.au.
